# Identification of Ferroptosis‐Related Gene in Age‐Related Macular Degeneration Using Machine Learning

**DOI:** 10.1002/iid3.70059

**Published:** 2024-12-16

**Authors:** Meijiang Zhu, Jing Yu

**Affiliations:** ^1^ Tongji University School of Medicine, Shanghai Tenth People's Hospital Shanghai China

**Keywords:** age‐related macular degeneration, ferroptosis, machine learning

## Abstract

**Background:**

Age‐related macular degeneration (AMD) is a major cause of irreversible visual impairment, with dry AMD being the most prevalent form. Programmed cell death of retinal pigment epithelium (RPE) cells is a central mechanism in the pathogenesis of dry AMD. Ferroptosis, a recently identified form of programmed cell death, is characterized by iron accumulation‐induced lipid peroxidation. This study aimed to investigate the involvement of ferroptosis in the progression of AMD.

**Methods:**

A total of 41 samples of AMD and 50 normal samples were obtained from the data set GSE29801 for differential gene expression analysis and functional enrichment. Differentially expressed genes (DEGs) were selected and intersected with genes from the ferroptosis database to obtain differentially expressed ferroptosis‐associated genes (DEFGs). Machine learning algorithms were employed to screen diagnostic genes. The diagnostic genes were subjected to Gene Set Enrichment Analysis (GSEA). Expression differences of diagnostic genes were validated in in vivo and in vitro models.

**Results:**

We identified 462 DEGs when comparing normal and AMD samples. The GO enrichment analysis indicated significant involvement in key biological processes like collagen‐containing extracellular matrix composition, positive cell adhesion regulation, and extracellular matrix organization. Through the intersection with ferroptosis gene sets, we pinpointed 10 DEFGs. Leveraging machine learning algorithms, we pinpointed five ferroptosis feature diagnostic genes: VEGFA, SLC2A1, HAMP, HSPB1, and FADS2. The subsequent experiments validated the increased expression of SLC2A1 and FADS2 in the AMD ferroptosis model.

**Conclusion:**

The occurrence of ferroptosis could potentially contribute to the advancement of AMD. SLC2A1 and FADS2 have demonstrated promise as emerging diagnostic biomarkers and plausible therapeutic targets for AMD.

## Graphical abstract

Through immune cell infiltration analysis, we observed an increased abundance of macrophages in AMD, and a transition from M2 to M1 polarization. Machine learning and experimental validation indicated an upregulation of SLC2A1 and FADS2 in the iron‐induced AMD model. These findings suggest that these two genes could serve as diagnostic markers and therapeutic targets for AMD.

## 1 Introduction

Age‐related macular degeneration (AMD) constitutes a major cause of irreversible blindness predominantly affecting individuals above 60 years in developed nations [1]. The development of AMD is influenced by a complex interplay of genetic factors and environmental exposures, including aging, smoking, oxidative stress, inflammation, sunlight exposure, and genetic predisposition [2]. AMD presents in two primary forms, dry AMD and wet AMD, with the former accounting for the majority (over 90%) of cases. The pathogenesis of dry AMD begins with the accumulation of drusen deposits beneath the retinal layers, leading to degeneration of retinal pigment epithelium (RPE) cells, impaired photoreceptor function, and eventual geographic atrophy (GA) [3]. Unfortunately, there are currently no effective medical or surgical treatments for GA, and the pathogenesis mechanisms of AMD remain elusive. Overall, AMD is a severe ocular disease with multifaceted etiology and a complex pathogenesis that necessitates further research.

Ferroptosis as a novel mode of regulated cell death relies on iron and lipid peroxides. Its distinguishing characteristics encompass heightened levels of free iron, lipid peroxide accumulation, and a unique cell demise process setting it apart from other forms of necrosis [4]. Central to this mechanism is the labile iron pool (LIP), which dynamically houses varying quantities of free iron. In this context, free iron reacts with hydrogen peroxide, ultimately yielding hydroxyl radicals and facilitating lipid peroxide accumulation [5, 6]. Recent investigations have associated ferroptosis with various diseases, including neurodegenerative disorders, myocardial infarctions, and ischemia‐reperfusion injuries [7]. Notably, the excessive accumulation of iron in the retina and choroid has been linked to AMD [8, 9]. Nevertheless, the precise involvement of ferroptosis‐associated genes in the diagnosis, prognosis, or therapeutic strategies for AMD necessitates further elucidation.

In this study, the investigation centered on the pathogenesis of AMD through a comprehensive analysis integrating gene expression disparities, particularly focusing on ferroptosis‐associated genes (DEFGs). Leveraging the Gene Expression Omnibus (GEO) database, a comparative analysis was conducted between normal and AMD samples to discern differential gene expression patterns. Through the intersection of differential gene sets with ferroptosis‐associated genes, distinct DEFGs were pinpointed. Subsequently, machine learning algorithms were employed to discern pivotal differential genes, enabling the classification of 41 AMD patients into distinct groups based on their DEFG profiles. In summation, this study contributes to the understanding of AMD's potential pathogenesis by elucidating disparities in gene expression, underscoring the significance of DEFGs, harnessing machine learning techniques, and delving into the intricate interplay between ferroptosis and AMD.

## 2 Materials and Methods

### 2.1 Databases and Data Collection

In this study, microarray data of the GSE29801 raw data sets [10] in the GEO database [11] were processed using the R program “GEOquery.” This data set included gene expression profiles from 142 AMD and 151 normal samples. Subsequently, 91 samples, comprising 50 normal eye tissues and 41 AMD‐affected eye tissues specifically from the Macular RPE‐choroid region, were selected for analysis (Table 1). The acquired microarray data underwent systematic preprocessing, involving probe‐to‐gene symbol alignment, removal of non‐matching probes, and application of a log_2_ transformation to ensure data consistency.

**Table 1:** Ninety‐one samples from GSE29801.

Sample GEO accession	Ocular disease	AMD classification	Tissue
GSM738433	Normal	Normal	Macular RPE‐choroid
GSM738435	Normal	Normal	Macular RPE‐choroid
GSM738437	Normal	Normal	Macular RPE‐choroid
GSM738439	Normal	Normal	Macular RPE‐choroid
GSM738441	Normal	Normal	Macular RPE‐choroid
GSM738443	Normal	Normal	Macular RPE‐choroid
GSM738445	Normal	Normal	Macular RPE‐choroid
GSM738447	Normal	Normal	Macular RPE‐choroid
GSM738450	Normal	Normal	Macular RPE‐choroid
GSM738452	Normal	Normal	Macular RPE‐choroid
GSM738453	Normal	Normal	Macular RPE‐choroid
GSM738455	Normal	Normal	Macular RPE‐choroid
GSM738457	Normal	Normal	Macular RPE‐choroid
GSM738459	Normal	Normal	Macular RPE‐choroid
GSM738461	Normal	Normal	Macular RPE‐choroid
GSM738464	Normal	Normal	Macular RPE‐choroid
GSM738466	Normal	Normal	Macular RPE‐choroid
GSM738469	Normal	Normal	Macular RPE‐choroid
GSM738471	Normal	Normal	Macular RPE‐choroid
GSM738472	Normal	Normal	Macular RPE‐choroid
GSM738474	Normal	Normal	Macular RPE‐choroid
GSM738476	Normal	Normal	Macular RPE‐choroid
GSM738478	Normal	Normal	Macular RPE‐choroid
GSM738480	Normal	Normal	Macular RPE‐choroid
GSM738483	Normal	Normal	Macular RPE‐choroid
GSM738484	Normal	Normal	Macular RPE‐choroid
GSM738486	Normal	Normal	Macular RPE‐choroid
GSM738488	Normal	Normal	Macular RPE‐choroid
GSM738491	Normal	Normal	Macular RPE‐choroid
GSM738494	Normal	Normal	Macular RPE‐choroid
GSM738495	Normal	Normal	Macular RPE‐choroid
GSM738497	Normal	Normal	Macular RPE‐choroid
GSM738498	Normal	Normal	Macular RPE‐choroid
GSM738500	Normal	Normal	Macular RPE‐choroid
GSM738503	Normal	Normal	Macular RPE‐choroid
GSM738505	Normal	Normal	Macular RPE‐choroid
GSM738506	Normal	Normal	Macular RPE‐choroid
GSM738508	Normal	Normal	Macular RPE‐choroid
GSM738510	Normal	Normal	Macular RPE‐choroid
GSM738511	Normal	Normal	Macular RPE‐choroid
GSM738512	Normal	Normal	Macular RPE‐choroid
GSM738514	Normal	Normal	Macular RPE‐choroid
GSM738515	Normal	Normal	Macular RPE‐choroid
GSM738516	Normal	Normal	Macular RPE‐choroid
GSM738518	Normal	Normal	Macular RPE‐choroid
GSM738520	Normal	Normal	Macular RPE‐choroid
GSM738521	Normal	Normal	Macular RPE‐choroid
GSM738523	Normal	Normal	Macular RPE‐choroid
GSM738525	Normal	Normal	Macular RPE‐choroid
GSM738527	Normal	Normal	Macular RPE‐choroid
GSM738529	AMD	Dry AMD	Macular RPE‐choroid
GSM738531	AMD	Dry AMD	Macular RPE‐choroid
GSM738533	AMD	MD1	Macular RPE‐choroid
GSM738535	AMD	MD1	Macular RPE‐choroid
GSM738537	AMD	MD1	Macular RPE‐choroid
GSM738539	AMD	Dry AMD	Macular RPE‐choroid
GSM738541	AMD	GA	Macular RPE‐choroid
GSM738543	AMD	Dry AMD	Macular RPE‐choroid
GSM738545	AMD	Dry AMD	Macular RPE‐choroid
GSM738547	AMD	Dry AMD	Macular RPE‐choroid
GSM738549	AMD	Dry AMD	Macular RPE‐choroid
GSM738551	AMD	CNV	Macular RPE‐choroid
GSM738553	AMD	Dry AMD	Macular RPE‐choroid
GSM738554	AMD	MD2	Macular RPE‐choroid
GSM738556	AMD	Clinical AMD diagnosis	Macular RPE‐choroid
GSM738558	AMD	CNV	Macular RPE‐choroid
GSM738560	AMD	GA/CNV	Macular RPE‐choroid
GSM738562	AMD	MD2	Macular RPE‐choroid
GSM738564	AMD	Dry AMD	Macular RPE‐choroid
GSM738566	AMD	Dry AMD	Macular RPE‐choroid
GSM738568	AMD	MD1	Macular RPE‐choroid
GSM738570	AMD	CNV	Macular RPE‐choroid
GSM738572	AMD	Clinical AMD diagnosis	Macular RPE‐choroid
GSM738574	AMD	Clinical AMD diagnosis	Macular RPE‐choroid
GSM738576	AMD	Dry AMD	Macular RPE‐choroid
GSM738578	AMD	GA/CNV	Macular RPE‐choroid
GSM738580	AMD	Dry AMD	Macular RPE‐choroid
GSM738582	AMD	Dry AMD	Macular RPE‐choroid
GSM738584	AMD	MD1	Macular RPE‐choroid
GSM738585	AMD	Clinical AMD diagnosis	Macular RPE‐choroid
GSM738587	AMD	CNV	Macular RPE‐choroid
GSM738589	AMD	Clinical AMD diagnosis	Macular RPE‐choroid
GSM738591	AMD	GA	Macular RPE‐choroid
GSM738593	AMD	MD1	Macular RPE‐choroid
GSM738595	AMD	Dry AMD	Macular RPE‐choroid
GSM738597	AMD	MD2	Macular RPE‐choroid
GSM738599	AMD	GA/CNV	Macular RPE‐choroid
GSM738601	AMD	Clinical AMD diagnosis	Macular RPE‐choroid
GSM738603	AMD	MD2	Macular RPE‐choroid
GSM738605	AMD	Dry AMD	Macular RPE‐choroid
GSM738607	AMD	Dry AMD	Macular RPE‐choroid

Additionally, a comprehensive set of 259 ferroptosis‐associated genes was sourced from the FerrDb V2 database [12] (http://www.zhounan.org/ferrdb/current/).

### 2.2 Differential Expression Analysis

First, DEG analysis was carried out on a collection of 41 AMD and 50 normal ocular samples. This analysis was conducted using the “limma” package [13] within the R programming environment. Distinctive gene expression patterns were identified, employing a dual threshold criterion of *p*‐value < 0.05 and |log_2_FC | > 0.263 (equivalent to a fold change of 1.2). These differential genes were effectively visualized through the implementation of volcano plots. Furthermore, to shed light on the biological significance and associated pathways of these DEGs, Gene Ontology (GO) enrichment analyses were carried out, employing the “clusterProfiler” package [14] in R.

Then, a Venn analysis was executed to assess the overlap between the DEGs and the ferroptosis‐associated genes. The genes present in the intersection of these two gene sets were considered as differentially expressed ferroptosis‐related genes (DEFGs). Box plots were generated to visualize the expression of DEFGs in the disease and control groups.

### 2.3 Machine Learning

We employed three distinct machine learning algorithms to pinpoint diagnostic genes based on the expression values of the DEFGs. The LASSO (Least Absolute Shrinkage and Selection Operator) algorithm was employed for the purpose of feature selection, with the objective of identifying pertinent genes while minimizing error rates [15]. Support Vector Machine with Recursive Feature Elimination (SVM‐RFE) was deployed to ascertain the optimal gene combination characterized by the lowest error rates and heightened accuracy [16]. The selection of the Random Forest (RF) algorithm was underpinned by its adaptability and accuracy in predicting continuous variables [17]. These analyses were executed utilizing the R packages “glmnet” [18], “e1071” [19] and “randomForest” [20] respectively. The genes identified at the intersection of these algorithmic outputs were recognized as crucial ferroptosis‐signatures diagnostic genes specific to AMD.

Subsequently, a multiple logistic regression was conducted using the “pROC” R package [21] to calculate the regression coefficients for each diagnostic gene. We downloaded three data sets, GSE99248 [22], GSE50195 [23], and GSE125564 [24], from the GEO database to serve as validation sets. Details of the genetic risk score are provided in Table 2. The diagnostic score was determined using the following formula:

$$\text{Riskscore}=\sum \beta_{\text{gene}}\times {\text{Exp}}_{\text{gene}}.$$

**Table 2:** Genetic risk scores for diagnostic genes.

name	OR (univariable)	OR (multivariable)	OR (final)
VEGFA	1.88 (1.36–2.61, *p* < 0.001)	1.95 (1.21–3.14, *p* = 0.006)	1.80 (1.16–2.79, *p* = 0.009)
SLC2A1	1.64 (1.24–2.17, *p* < 0.001)	0.69 (0.43–1.11, *p* = 0.128)	0.71 (0.45–1.11, *p* = 0.135)
HAMP	1.50 (1.17–1.92, *p* = 0.001)	1.34 (0.99–1.82, *p* = 0.060)	1.34 (0.99–1.81, *p* = 0.054)
HSPB1	0.72 (0.58–0.88, *p* = 0.002)	0.68 (0.43–1.08, *p* = 0.102)	0.60 (0.44–0.82, *p* = 0.001)
XBP1	0.64 (0.48–0.85, *p* = 0.002)	0.98 (0.60–1.60, *p* = 0.939)	
HERPUD1	0.66 (0.51–0.86, *p* = 0.002)	0.95 (0.58–1.55, *p* = 0.841)	
FADS2	1.57 (1.18–2.10, *p* = 0.002)	2.00 (1.06–3.77, *p* = 0.032)	2.26 (1.53–3.34, *p* < 0.001)
ATF3	0.82 (0.69–0.98, *p* = 0.028)	0.87 (0.66–1.15, *p* = 0.327)	
SCD	1.25 (1.01–1.55, *p* = 0.038)	1.09 (0.71–1.67, *p* = 0.703)	

Among the formula, the symbol *β*_gene_ signifies the regression coefficient derived from LASSO regression analysis of corresponding diagnostic gene, while Exp_gene_ denotes the expression of the gene within each distinct sample.

### 2.4 GSEA Analysis for Ferroptosis Feature Diagnostic Genes

Gene Set Enrichment Analysis (GSEA) was conducted using the MSigDB v7.1 [25] database, specifically employing the c2.cp.kegg.v7.4.symbols.gmt file as the background for enrichment analysis. The gene expression profiles of AMD patients were subjected to GSEA to identify KEGG pathways that exhibited significant associations with the diagnostic genes. Enriched gene sets meeting a *p*‐value < 0.05 were selected and presented for further analysis.

### 2.5 In Vitro Analyses

To substantiate the diagnostic relevance of five ferroptosis feature genes in AMD, a combination of in vivo and in vitro experiments was executed. We induced the human RPE cell line ARPE‐19 using sodium iodate (SI) and ferrous ammonium citrate (FAC). SI intervention is a classical method for establishing an AMD model [26], while FAC, an iron‐containing salt, is used to induce ferroptosis [27]. The FAC group was treated with 50 mg/mL ferric ammonium citrate, and the SI group was treated with 2.5 mM SI for 48 h. Quantitative polymerase chain reaction (qPCR) was employed to measure the expressions of VEGFA, SLC2A1, HAMP, HSPB1, and FADS2 genes. The process involved the extraction of total RNA, synthesis of complementary DNA (cDNA), and utilization of SYBR Green Premix for qPCR analysis. Statistical evaluation was performed through independent‐sample t‐test at a significance level of *p* < 0.05. The primers used in the experiments were synthesized by Sangon Biotech (Shanghai, China) and are listed as follows: human GAPDH (F:5′‐GGACCTGACCTGCCGTCTAGAA‐3′; R:5′‐GGTGTCGCTGTTGAAGTCAGAG‐3′), human VEGFA (F:5′‐AGGGCAGAATCATCACGAAGT‐3′; R:5′‐AGGGTCTCGATTGGATGGCA‐3′), human SLC2A1(F:5′‐GGCCAAGAGTGTGCTAAAGAA‐3′; R:5′‐ACAGCGTTGATGCCAGACAG‐3′), human HAMP (F:5′‐CTGACCAGTGGCTCTGTTTTC‐3′; R:5′‐GAAGTGGGTGTCTCGCCTC‐3′), human FADS2 (F:5′‐TGACCGCAAGGTTTACAACAT‐3′; R:5′‐AGGCATCCGTTGCATCTTCTC‐3′), human HSPB1(F:5′‐ACGGTCAAGACCAAGGATGG‐3′; R:5′‐AGCGTGTATTTCCGCGTGA‐3′).

Additionally, Western Blot analysis was conducted to validate the gene expression levels. Following PBS washing, cells were lysed with RIPA buffer containing a protease inhibitor cocktail (Sigma Aldrich). Standard procedures were adhered to during subsequent protein extraction. Each channel of 12.5% polyacrylamide gels received 30 μg of protein extracts. Post‐electrophoresis, proteins were transferred to nitrocellulose membrane (Whatman, UK). Subsequently, the membrane was blocked using 5% nonfat milk or 5% BSA. Following blocking, the membrane was subjected to overnight incubation with the primary antibody FADS2 (Proteintech, 28034‐1‐AP) and SLC2A1 (Proteintech, 21829‐1‐AP) at 4°C and later exposed to a specific secondary antibody at room temperature for 1 h. Finally, protein bands were scanned using the Odyssey imaging system (LICOR). Band intensities were quantified utilizing the Image Studio System (Version 5.2.5).

### 2.6 In Vivo Analyses

C57BL/6J male mice aged 6 to 8 weeks were sourced from Beijing Vital River Laboratory Animal Technology for experimental purposes. SI was administered intraperitoneally at a dose of 40 mg/kg to establish a mouse model of AMD [26]. After 7 days, mice were humanely euthanized, and their eyes were carefully extracted for subsequent RPE flatmount and eye section preparation. Immunofluorescence staining using ZO‐1 antibody (Proteintech, 21773‐1‐AP), was applied to RPE flatmount sections. Further analyses involved paraffin embedding, hematoxylin and eosin (HE) staining, and immunofluorescence (IF) staining on eye specimens. The animal experiments adhered to the ARRIVE guidelines and received ethical approval from the Ethics Committee of Shanghai Tenth People's Hospital (Approval No. SHDSYY‐2023‐1902).

### 2.7 Statistical Analysis

The experimental procedures were replicated on a minimum of three separate occasions to ensure the robustness of the findings. For statistical assessments, R version 4.21 and GraphPad Prism 8 were employed. Two‐group comparisons were subjected to Student's *t*‐test, while more than three groups were analyzed using one‐way ANOVA. The significance threshold was set at *p* < 0.05, aligning with accepted standards for statistical significance in the field.

## 3 Results

### 3.1 Identification of DEGs and Functional Enrichment Analysis

**Figure 1:**
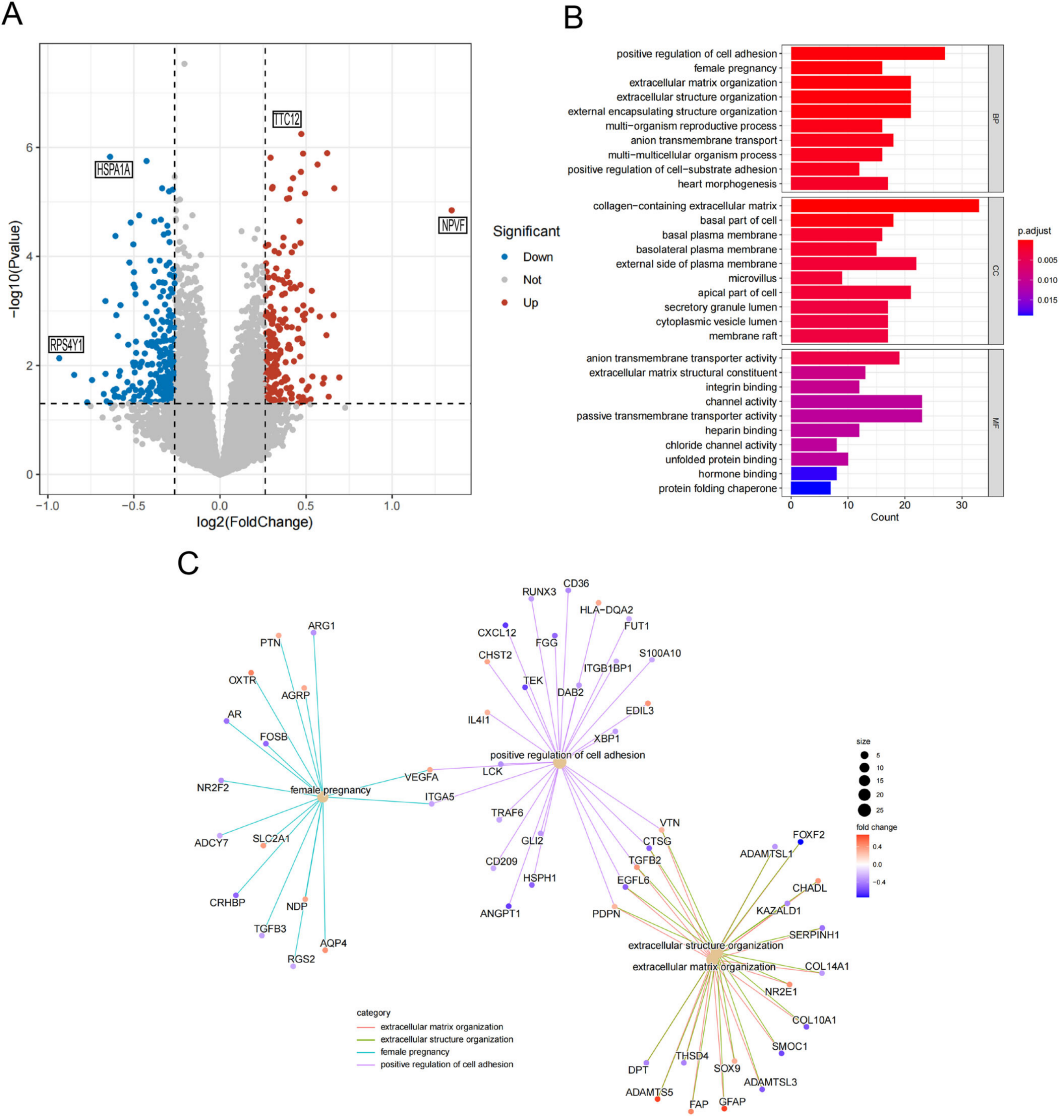
Identification and enrichment analysis of DEGs. (A) The volcano plot illustrating the 462 DEGs. (B) The GO barplot enrichment analysis of DEGs. (C) The GO network enrichment analysis of DEGs.

A total of 462 DEGs were identified between AMD and normal samples using the GSE29801 data set, with 211 genes upregulated and 251 genes downregulated in the disease condition. Visual representation of DEG distribution was achieved through a volcano plot (Figure 1A). Subsequent to conducting GO enrichment analyses, facilitated by the cluster Profiler software package within the R environment, the foremost Gene Ontology categories within biological processes (BPs), cellular components (CCs), and molecular functions (MFs) were discerned as encompassing the promotion of cell adhesion, collagen−containing extracellular matrix, and transmembrane transporter activity, respectively (Figure 1B). Moreover, we also demonstrated positive regulation of cell adhesion, female pregnancy, extracellular matrix organization, extracellular structure organization are the top 4 terms according to p‐values, and their corresponding pathways and genes association were visualized in a network graph (Figure 1C). These insightful findings provide enhanced understanding into the functional implications of the DEGs in the context of AMD.

### 3.2 Identification of the Ferroptosis‐Related DEGs

**Figure 2:**
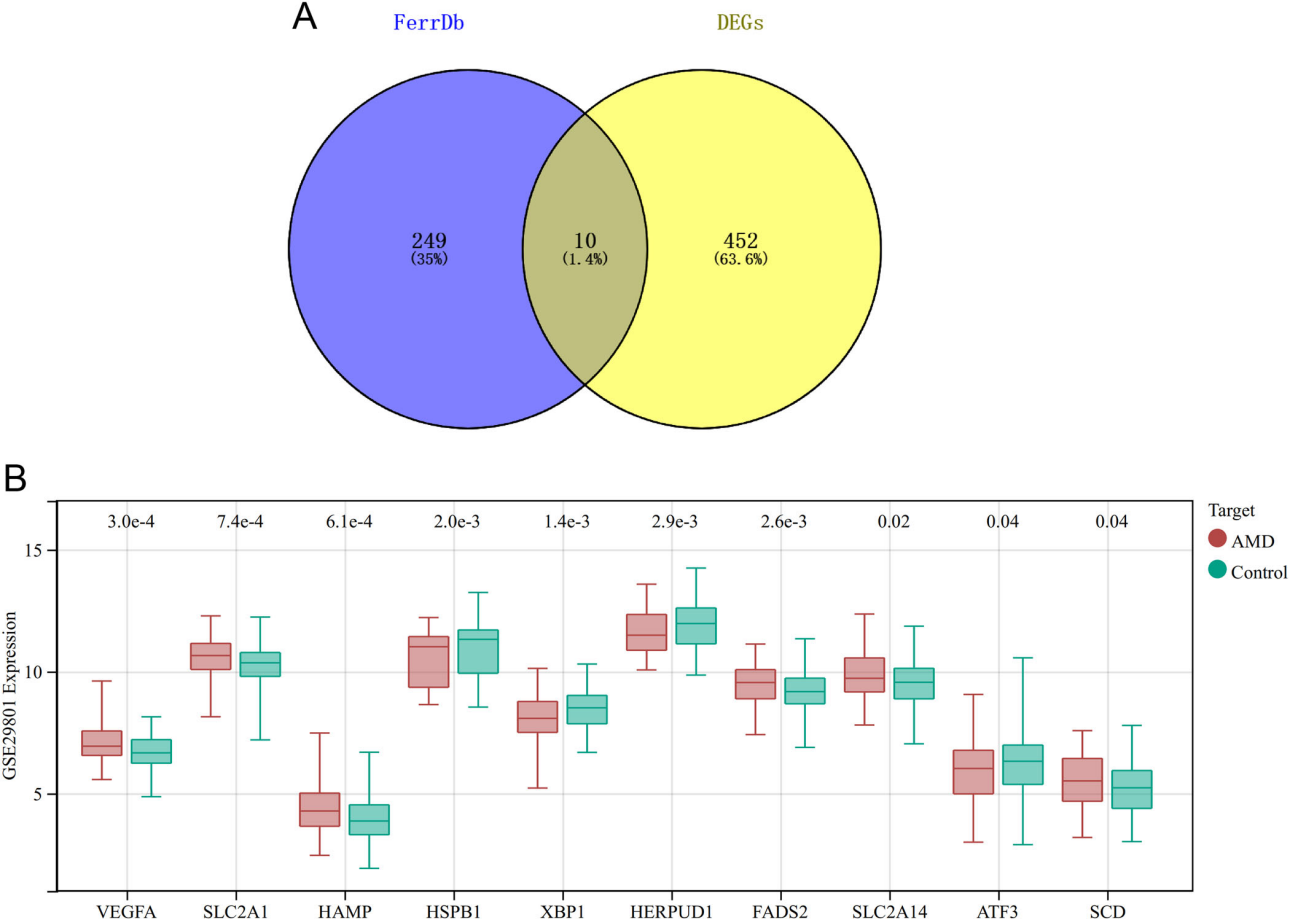
Identification of DEFGs. (A) The Venn diagram illustrates the genes that are common between DEGs and ferroptosis‐related genes. (B) Box plots provide an overview of the expression levels of DEFGs in AMD patients.

A Venn analysis was conducted to assess the overlap between the DEGs and ferroptosis‐related genes. This analysis identified 10 genes referred to as differentially expressed ferroptosis‐related genes (DEFGs). These DEFGs encompass VEGFA, SLC2A1, HAMP, HSPB1, XBP1, HERPUD1, FADS2, SLC2A14, ATF3, and SCD (Figure 2A). The differential boxplots visually represent the expression levels of these DEFGs in both the disease and control groups, offering a concise portrayal of the gene expression disparities between these two groups (Figure 2B). These results identified the involvement of ferroptosis‐associated genes in the pathogenesis of AMD.

### 3.3 Identification of the Diagnostic Ferroptosis Feature Genes Via Machine Learning

**Figure 3:**
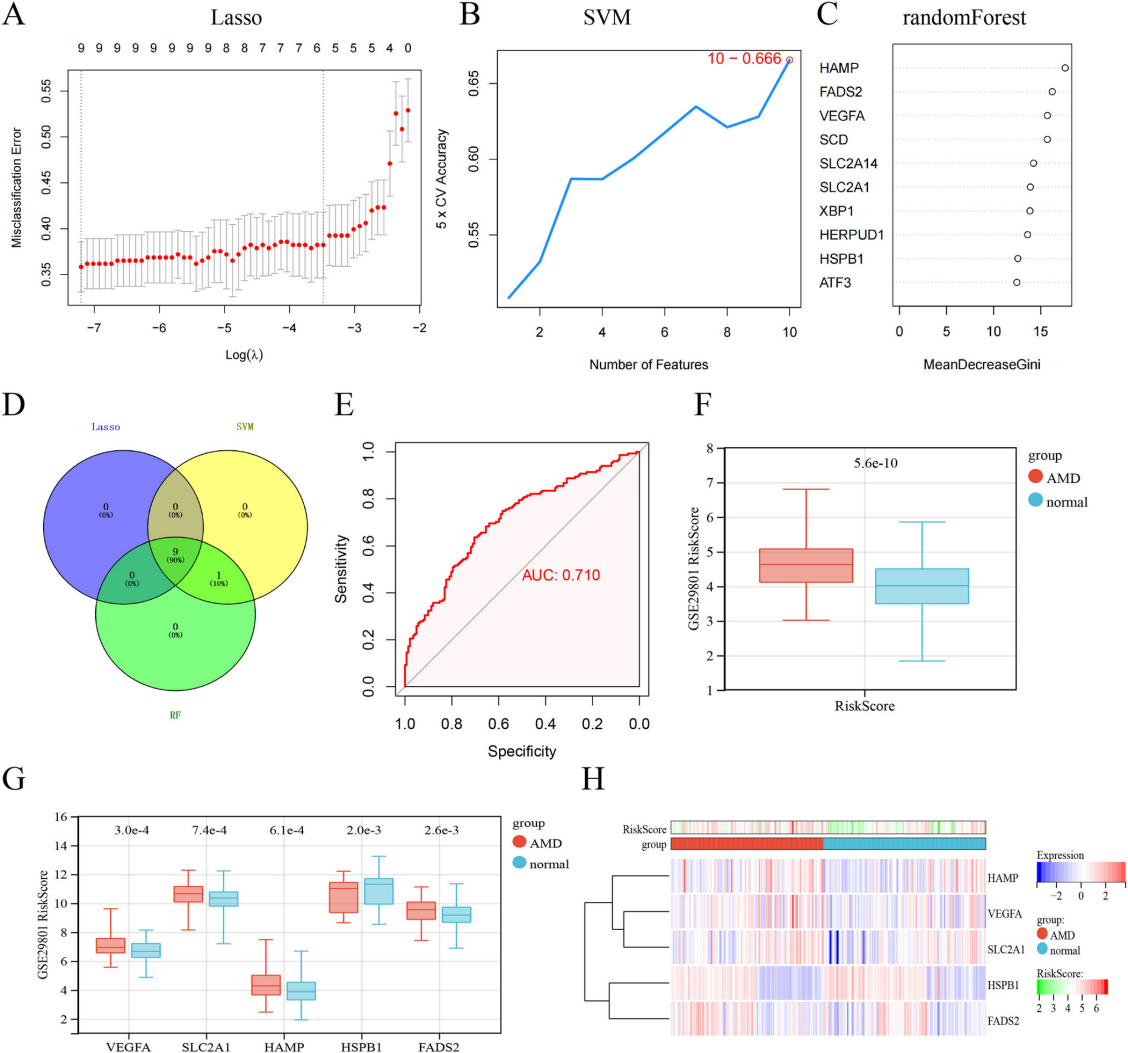
Machine learning in the identification of ferroptosis‐signatures diagnostic genes. (A–C) identify ferroptosis feature genes using LASSO regression, SVM, and RF algorithm. (D) The Venn diagram shows the overlap of candidate genes between the above three algorithms. (E) ROC curve of ferroptosis‐signatures in AMD diagnosis. (F, G) Box plots of RiskScores in the Normal and AMD groups. (H) Clustered heatmap of ferroptosis‐signatures diagnostic genes expression levels.

Using the gene expression profiles of the 10 DEFGs, a combination of LASSO regression, SVM‐RFE, and RF algorithms was employed to identify ferroptosis feature genes in AMD samples (Figure 3A–C). Nine genes (VEGFA, SLC2A1, HAMP, HSPB1, XBP1, HERPUD1, FADS2, ATF3 and SCD) were selected as the feature genes (Figure 3D). Subsequently, a multivariable logistic regression analysis model was conducted for diagnostic purposes, ultimately comprising five key genes (HAMP, HSPB1, VEGFA, SLC2A1, and FADS2). Evaluation of the model using ROC curve analysis revealed an AUC value exceeding 0.7, indicating favorable predictive performance for the disease (Figure 3E). Box plots were generated to visualize the distribution of RiskScores in the normal and AMD groups, demonstrating significantly higher RiskScores in the AMD group compared to the normal group, thus reinforcing the accuracy of the model (Figure 3F). The results indicated that VEGFA, SLC2A1, HAMP, and FADS2 were upregulated, while HSPB1 was downregulated in AMD compared to control samples (Figure 3G). Additionally, a heatmap further visualized distinct gene expression patterns across individual samples (Figure 3H).

### 3.4 GSEA Analysis for Five Diagnostic Ferroptosis Feature Genes

**Figure 4:**
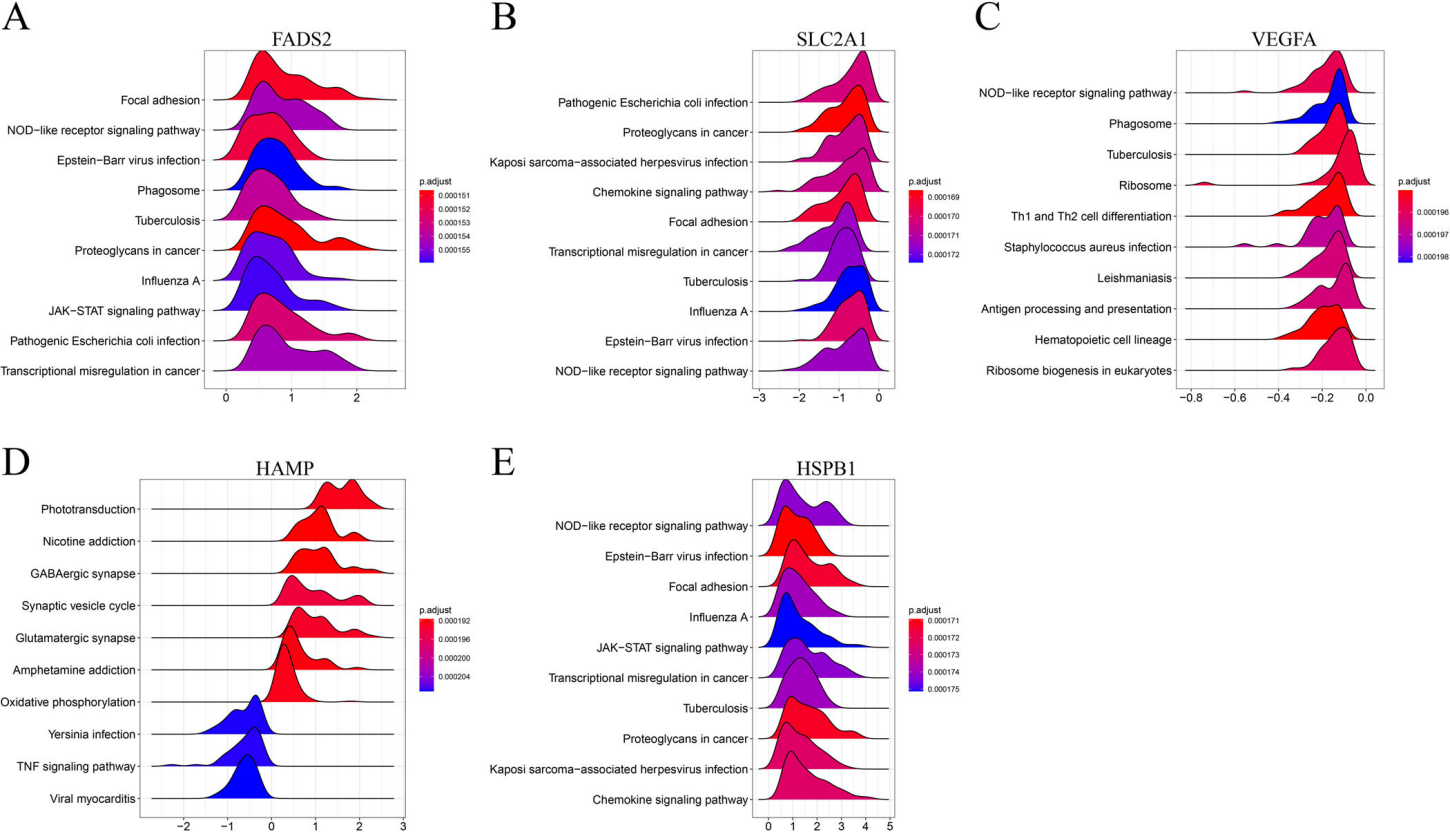
GSEA and small molecule drug prediction analysis. (A–G) GSEA investigation of FADS2, HAMP, HSPB1, SLC2A1, and VEGFA.

The GSEA algorithm was employed to ascertain the differential regulatory pathways between the high and low expression groups of the final five diagnostic genes, aiming to identify the activated signaling pathways in AMD by selecting pathways that exhibited a close association with the elevated expression of hub genes. Gene sets exhibiting enrichment with a *p*‐value < 0.05 were selected, and a ridge plot was generated to display the top ten pathways, ordered by their respective *p* values (Figure 4A–E). The results indicate that the diagnostic genes are predominantly enriched in the focal adhesion pathway and NOD‐like receptors (NLRs), which are involved in inflammation and immune‐related signaling pathways.

### 3.5 Validation of DEGs In Vitro

**Figure 5:**
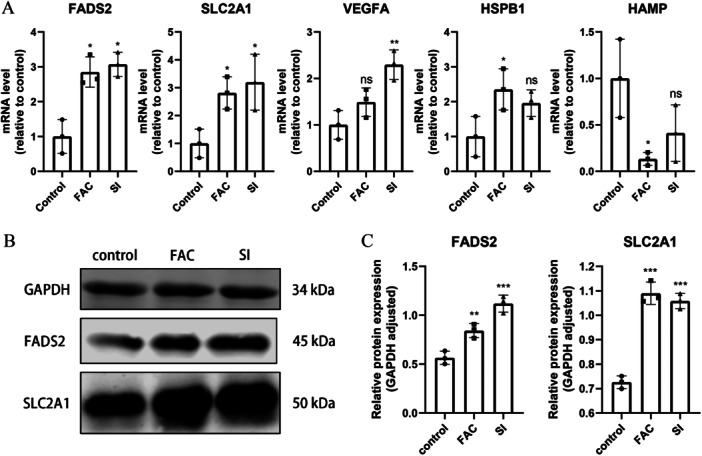
Expression levels of ferroptosis‐signature diagnostic genes in the ARPE‐19 cell line (A) mRNA expression of FADS2, HAMP, HSPB1, SLC2A1, and VEGFA. (B) Protein expression of FADS2 and SLC2A1.ns, no significance; *p < 0.05; **p < 0.01; ***p < 0.001.

The RT‐qPCR analysis revealed upregulation of FADS2 and SLC2A1 in both the FAC and SI experimental groups (Figure 5A). Considering this finding, we selected these two genes for further experimental validation. The Western blot results corroborated the RT‐qPCR findings, indicating congruency between the two techniques (Figure 5B).

### 3.6 Expression of FADS2 and SLC2A1 in an AMD Animal Model

**Figure 6:**
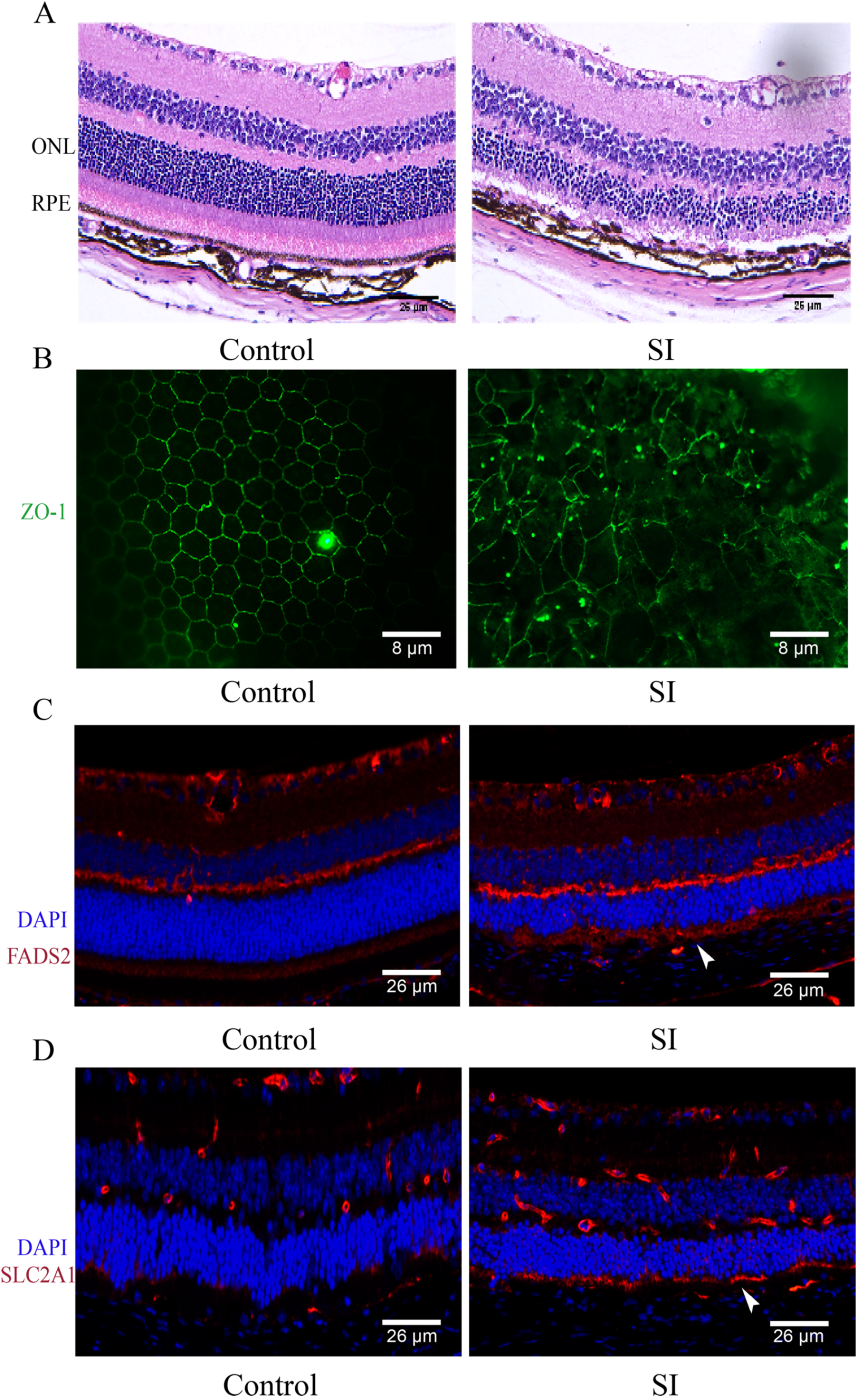
Validation of FADS2 and SLC2A1 expression in an AMD animal model. (A) HE staining shows disruption of the outer nuclear layer and loss of the RPE layer. (B) ZO‐1 staining indicates damage to the tight junctions of RPE cells. (C, D) IF staining shows high expression of FADS2 and SLC2A1 in the RPE area (indicated by arrowheads).

After intraperitoneal injection of SI for 7 days, retinal‐RPE tissues were collected for HE and ZO‐1 staining. HE results showed significant retinal degenerative changes, including a marked thinning of the outer nuclear layer, atrophy of the photoreceptor inner and outer segments, and obvious disorganization of the RPE layer (Figure 6A). ZO‐1 staining indicated morphological alterations in RPE cells, characterized by increased cell area and disrupted hexagonal structure, with evidence of tight junction breakdown in some areas (Figure 6B). These observations indicate that SI successfully induced an AMD mouse model. Furthermore, IF analysis demonstrated elevated expression levels of FADS2 and SLC2A1 in the RPE region of the SI group compared to the control group (Figure 6C,D).

## 4 Discussion

The multifaceted nature of AMD contributes to its challenging curability. The molecular mechanisms underlying this affliction remain complex and not completely understood. Presently, treatment for wet AMD involves intravitreal administration of anti‐VEGF agents, while effective diagnostic and therapeutic strategies for dry AMD are yet to be established. Ongoing endeavors for dry AMD treatment encompass corticosteroids, antioxidants, and neuroprotective agents, however, these modalities are still in their preliminary stages and have some adverse effects [28]. Therefore, there is a pressing need to investigate novel avenues for diagnosing and managing AMD. Our investigation establishes a correlation between ferroptosis and AMD pathogenesis, employs bioinformatics analyses to discern potential key genetic determinants, and explores potential therapeutic targets.

We investigated the gene expression profiles of both normal controls and individuals with AMD using the GEO database, identifying 462 DEGs. The GO enrichment analysis highlighted significant involvement in biological processes such as collagen‐containing extracellular matrix composition, positive regulation of cell adhesion, and extracellular matrix organization. The extracellular matrix (ECM) is a network of proteins, fibers, and molecules that supports cells and provides signaling [29]. It serves as a foundation for RPE adherence and interaction, maintaining integrity and function. Within the retinal region, the ECM influences RPE processes such as adhesion, migration, and nutrient exchange with nearby blood vessels, crucial for retinal health [30]. Previous research has provided evidence demonstrating that perturbations in the integrity and organization of the ECM within the retinal region correlate with compromised RPE function observed in AMD [31]. This pathological process is further exacerbated by factors such as inflammation, complement activation, and the participation of immune cells [32, 33].

Several studies have indicated heightened apoptotic cell death among individuals with AMD [34]. Meanwhile, ferroptosis, a novel programmed cell death mechanism [6, 7], necessitates further exploration into its regulatory implications across various disorders and underlying mechanisms. Consequently, we aimed to enhance our comprehension of ferroptosis‐related gene functions in AMD phenotyping. An initial step involved comparing regulatory expressions of DEFGs in ocular tissues from healthy individuals and AMD patients. The downregulated DEFGs in AMD patients compared to healthy controls underscored their pivotal role in AMD pathogenesis. Employing machine learning classifiers and multivariable logistic regression, we identified five key genes (HAMP, HSPB1, VEGFA, SLC2A1, and FADS2). Correlations among these hub DEFGs were examined, revealing compelling evidence of synergistic or antagonistic interactions in AMD patients. Importantly, VEGFA contributes to abnormal vascular growth and retinal leakage, exacerbating AMD progression [35]. FADS2 influences lipid metabolism and lipid‐related processes relevant to AMD [36]. SLC2A1 modulates glucose availability in the RPE, influencing oxidative stress‐mediated AMD pathology [37]. HAMP, encoding hepcidin, impacts iron regulation and AMD‐associated inflammation [38]. HSPB1's role in cellular stress responses and oxidative damage protection has implications for retinal health and disease trajectory [39]. However, documentation of these genes' significance in AMD is limited, necessitating further research.

Previous studies have reported that SI intervention exerts ferroptotic effects in AMD models [26], and FAC has also been used to induce ferroptosis [27]. The RT‐qPCR results demonstrated an upregulation of both FADS2 and SLC2A1 in both the FAC and SI cell models. This observation was further validated by IF analysis. FADS2's involvement in iron‐induced cell death is through its regulation of lipid peroxide accumulation. Its function in lipid metabolism, particularly the conversion of polyunsaturated fatty acids (PUFAs) into bioactive lipid mediators, suggests a potential influence on cellular membrane lipid composition and oxidative stress, consequently affecting susceptibility to ferroptosis [40]. This connection becomes more relevant in conditions like cancer [41] and neurodegenerative disorders [42], where FADS2's interaction with altered lipid profiles and oxidative stress aligns with the initiation of ferroptosis. In parallel, SLC2A1's role in glucose uptake, especially in glucose‐sensitive tissues like the retina, holds significance due to its effects on energy metabolism and NADPH generation, critical for oxidative stress mitigation. This connects SLC2A1 to ferroptosis through metabolic and redox pathways [43]. However, the precise regulatory roles of FADS2 and SLC2A1 in ferroptosis and their implications in the context of AMD necessitate further investigation to provide comprehensive insights. Our experimental validation has shown elevated expression of FADS2 and SLC2A1 in the RPE of AMD mouse models, potentially offering novel therapeutic targets for future AMD treatment strategies.

It is imperative to acknowledge the limitations of this study. Firstly, most of our findings are based on comprehensive bioinformatics analyses, highlighting the need for further confirmation through clinical trials. Additionally, the validation studies were conducted in cell cultures and SI models, the relevance of which to human AMD pathogenesis remains unclear. In future studies, we will utilize ocular tissues from donor eyes with and without the disease for further verification. Furthermore, while we identified ferroptosis‐related diagnostic genes involved in AMD progression, whether these diagnostic genes influence susceptibility to ferroptosis requires additional investigation. Although this study provides valuable insights, addressing these limitations is crucial. Incorporating clinical validation, expanding sample sizes, and conducting in‐depth mechanistic studies will significantly enhance the robustness and impact of the findings.

## Author Contributions

**Meijiang Zhu:** conceptualization, data curation, methodology, resources, software, validation, writing–original draft, writing–review and editing. **Jing Yu:** validation.

## Ethics Statement

This study involves animal subjects and was approved by Ethics Committee of Shanghai 10th People's Hospital. The ID number is SHDSYY‐2023‐1902.

## Consent

Not applicable.

## Conflicts of Interest

The author declares no conflicts of interest.

## Data Availability

The data sets generated and analyzed during the current study are available in the GSE29801 raw data sets, https://www.ncbi.nlm.nih.gov/geo/query/acc.cgi?acc=GSE29801.
